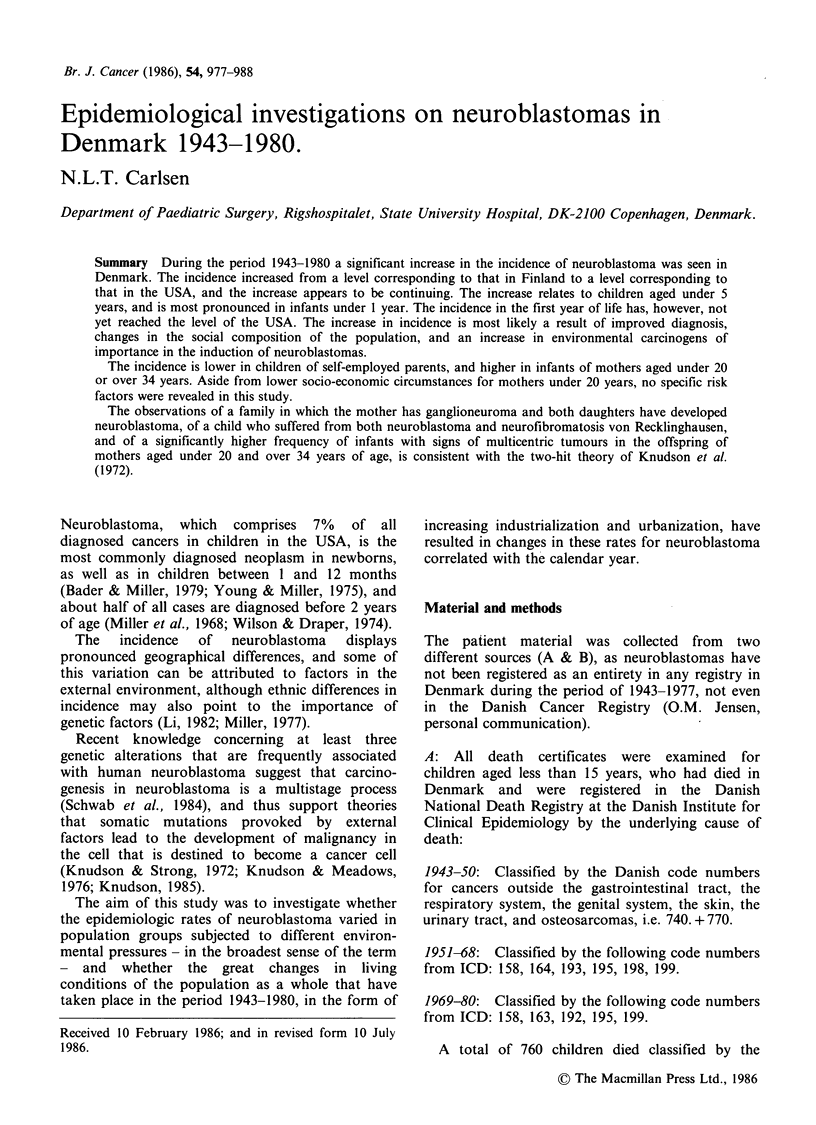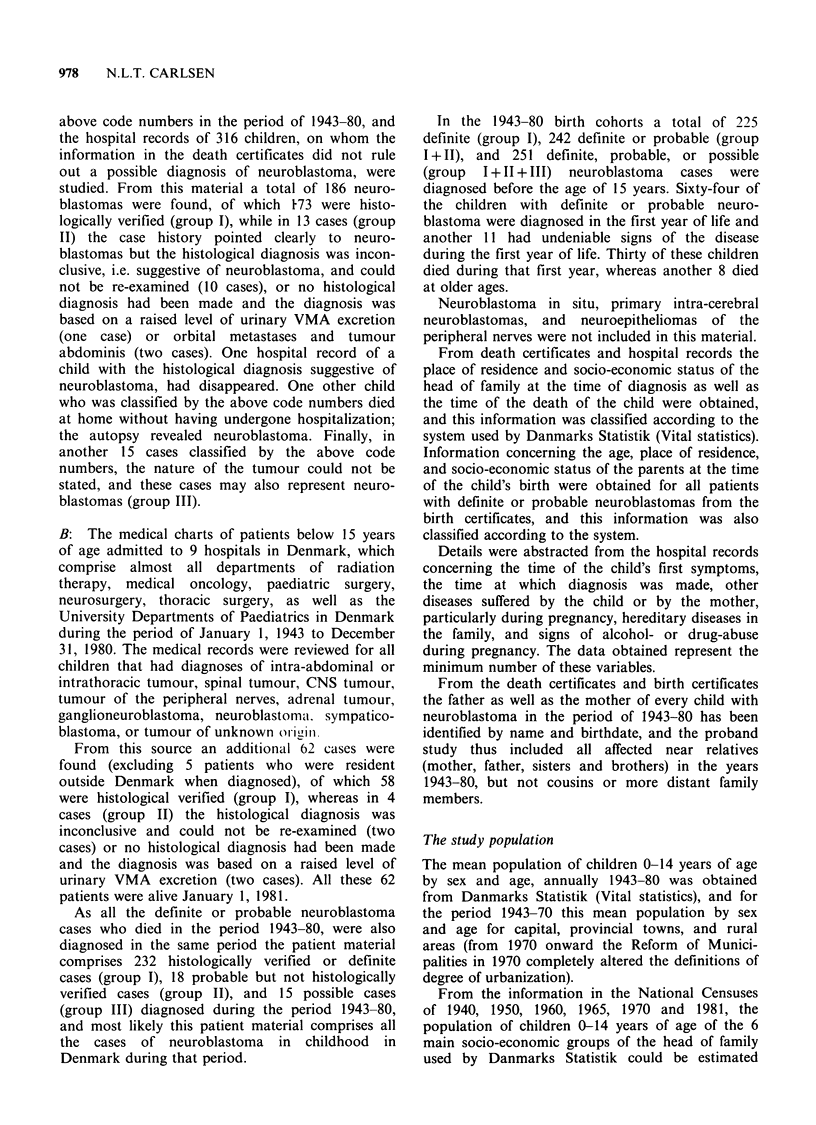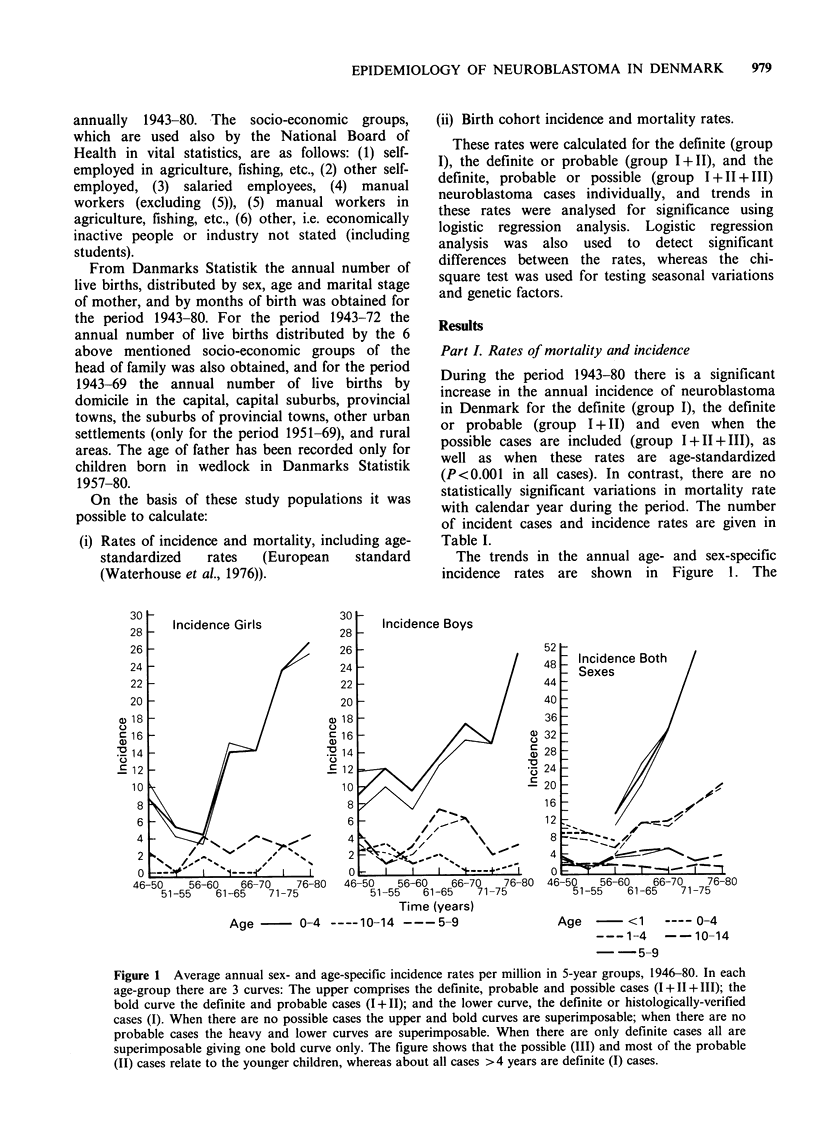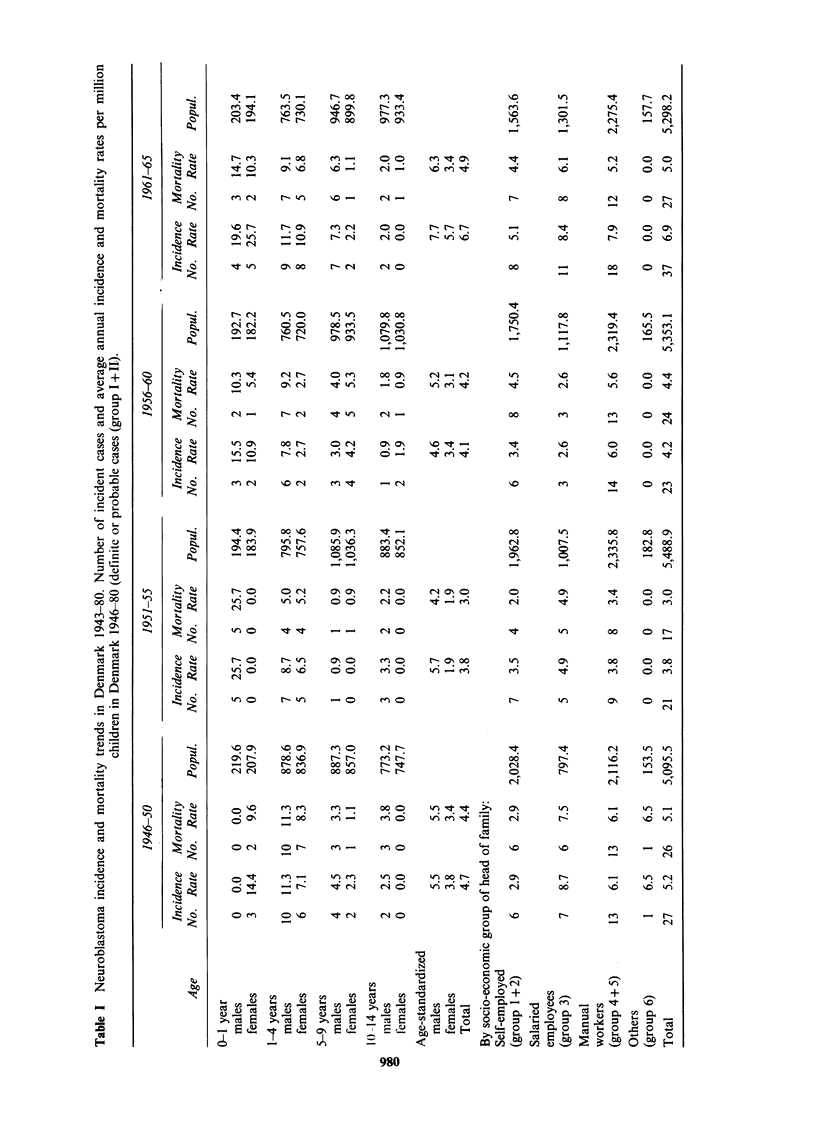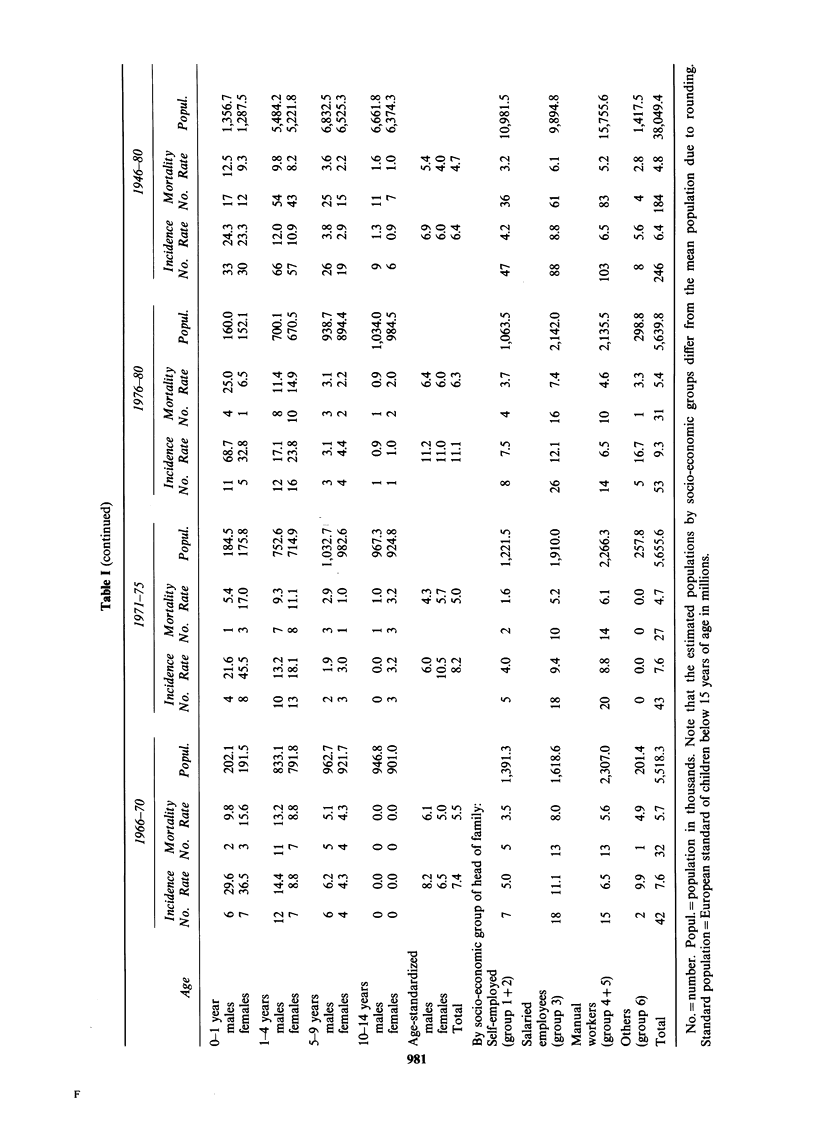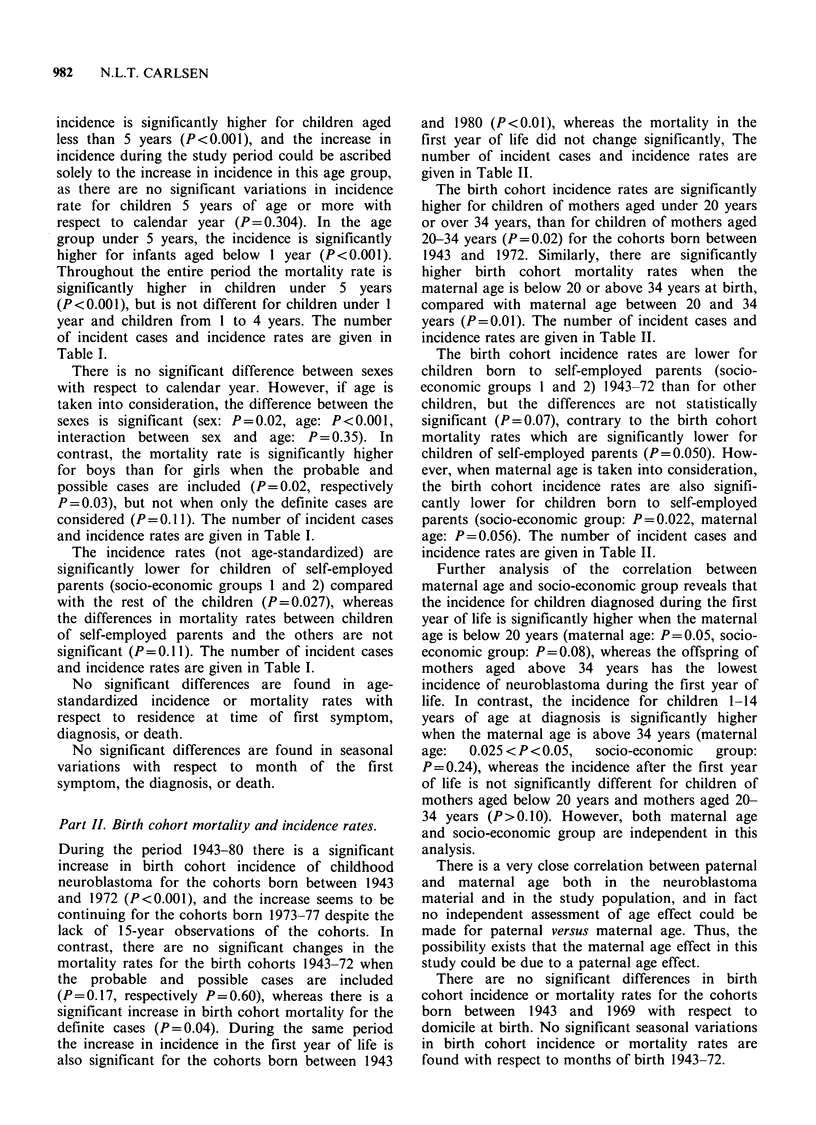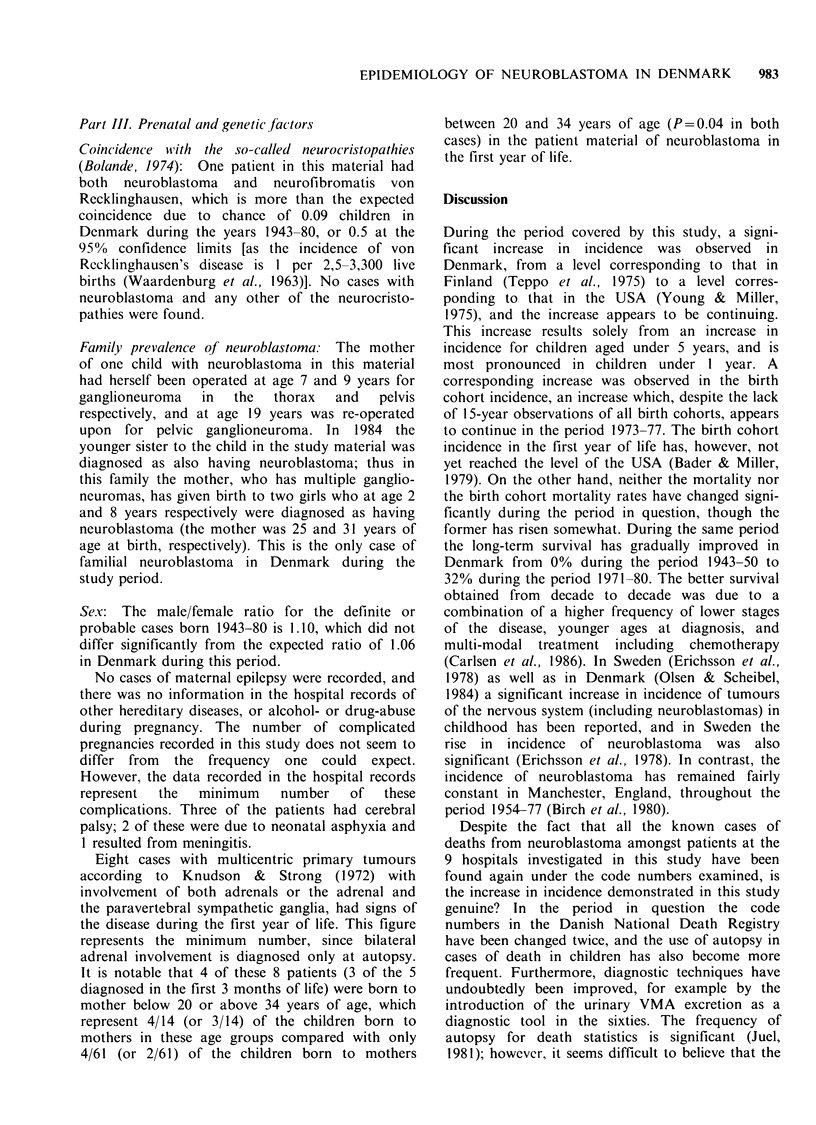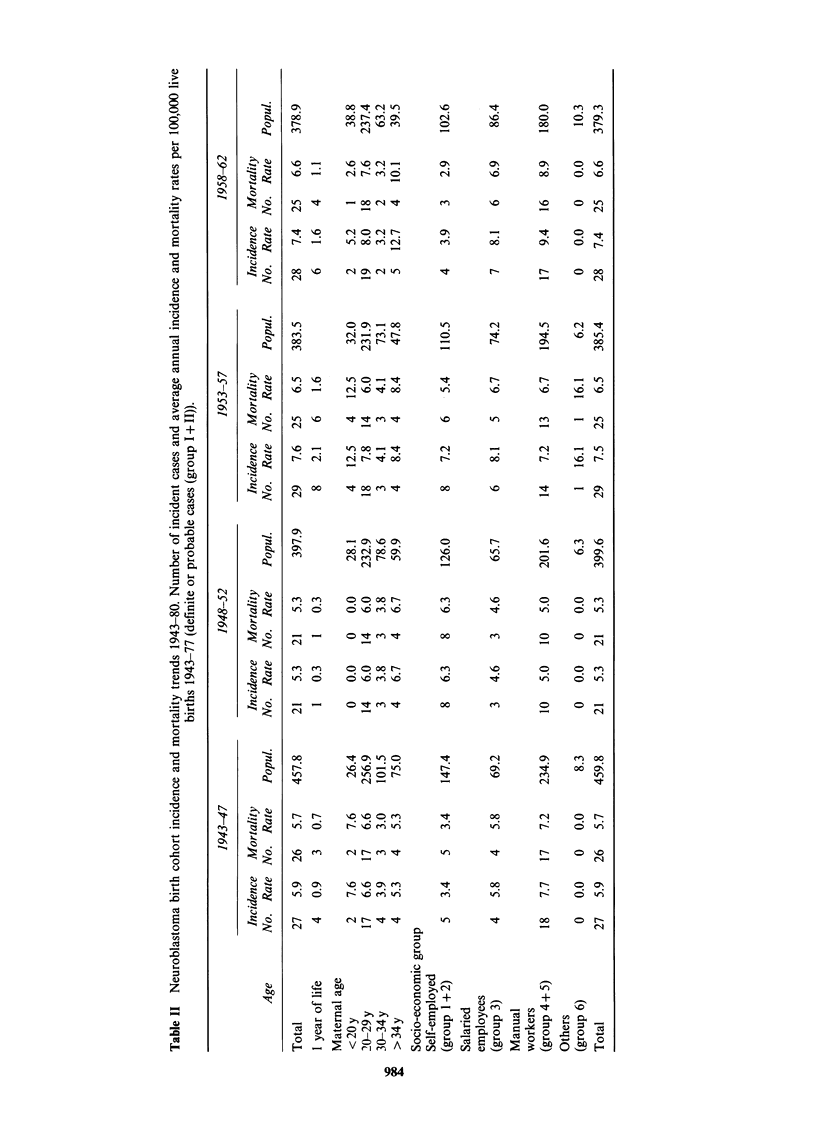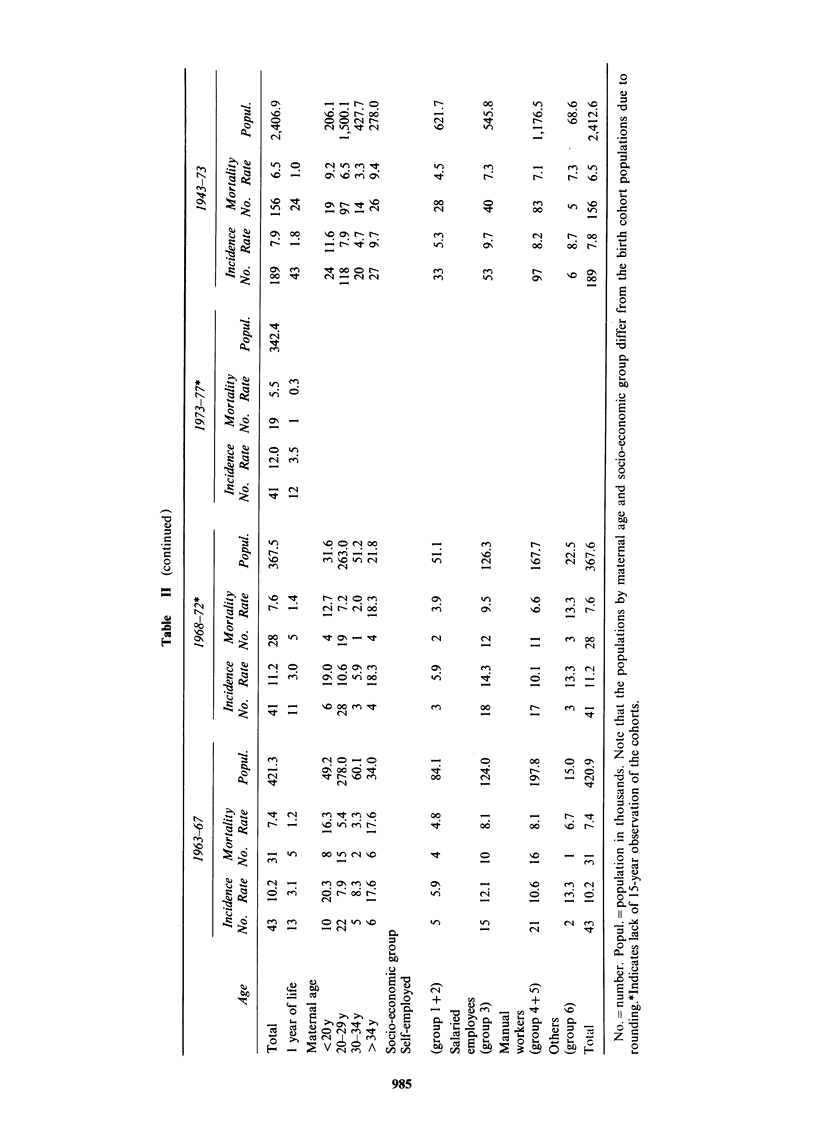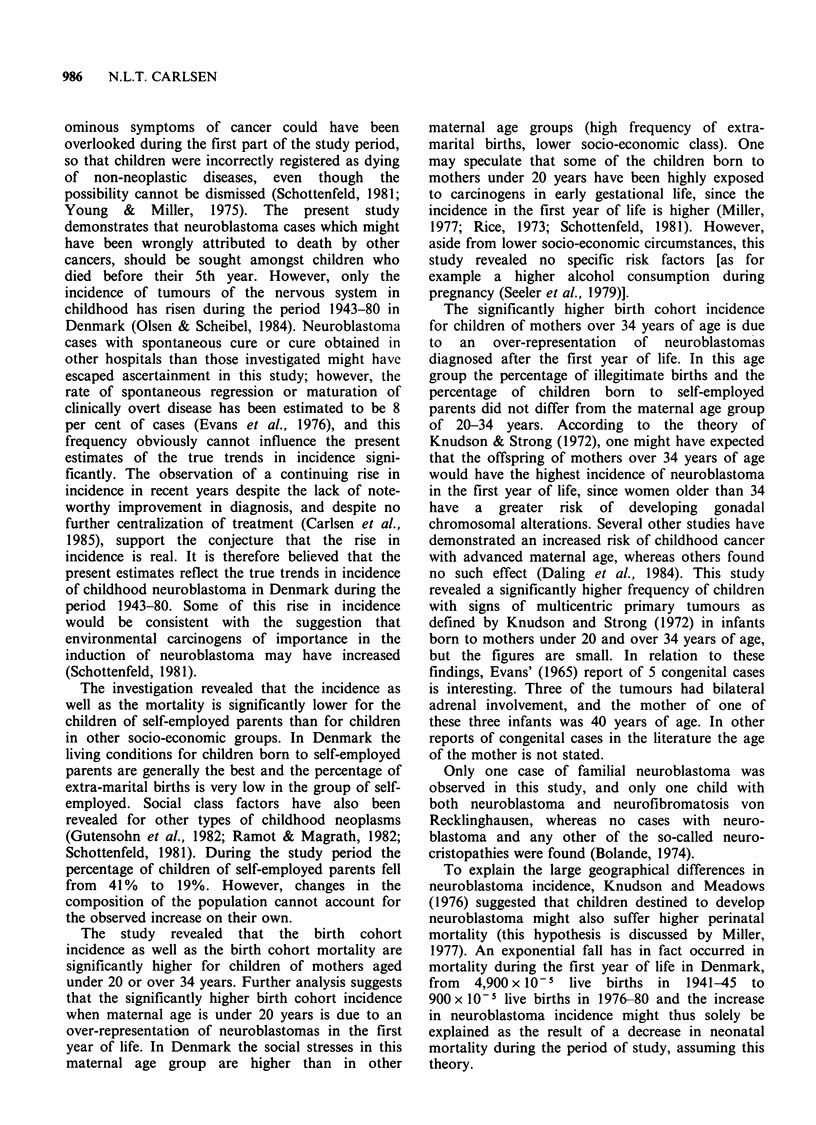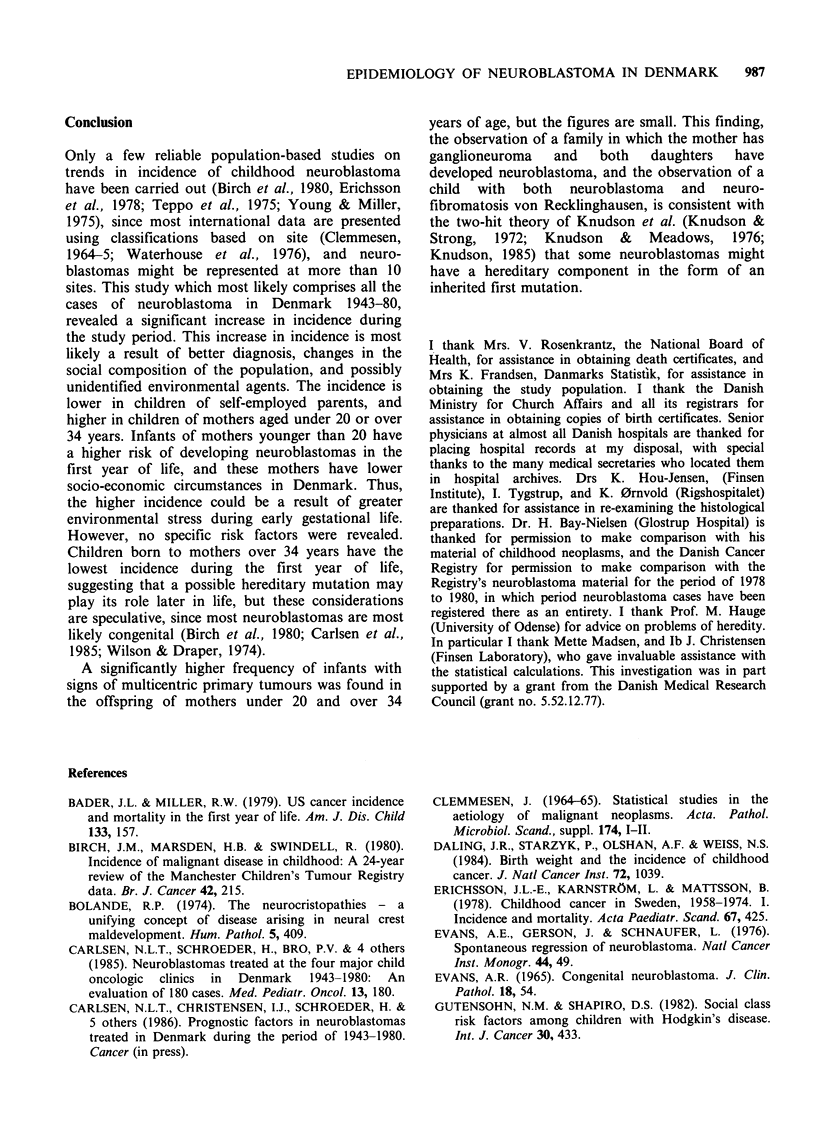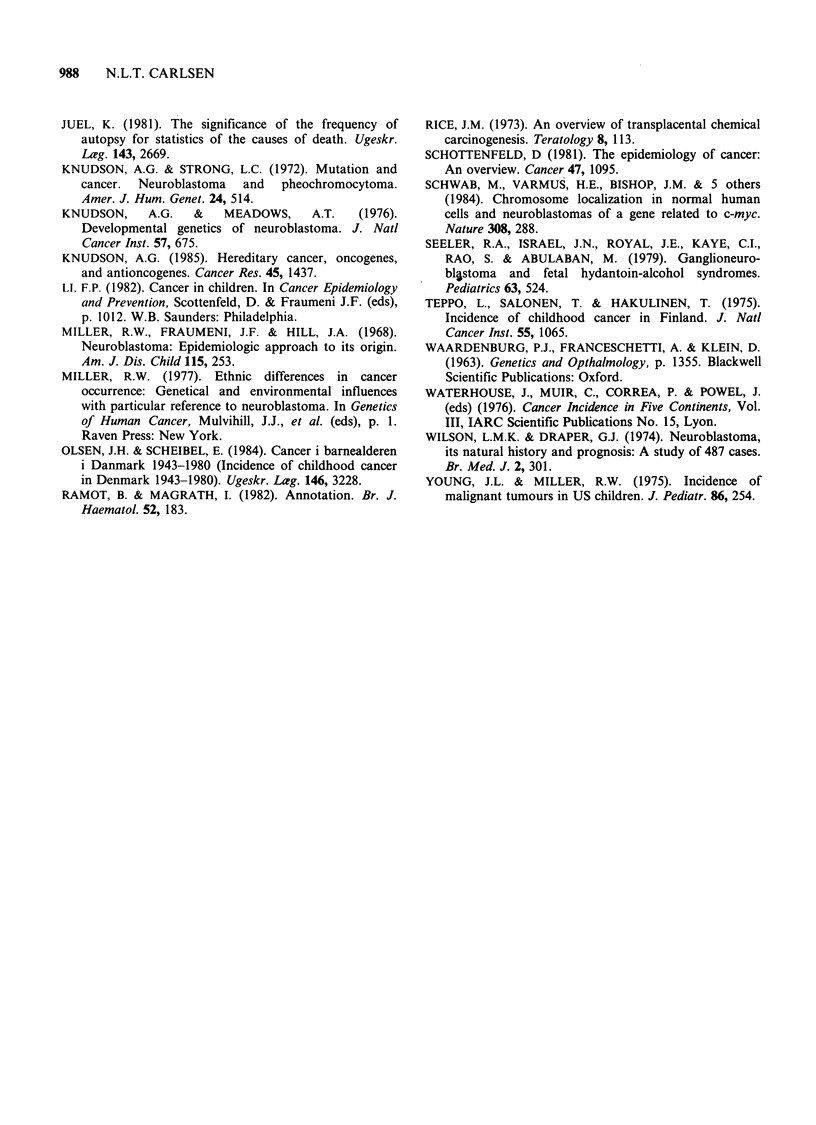# Epidemiological investigations on neuroblastomas in Denmark 1943-1980.

**DOI:** 10.1038/bjc.1986.270

**Published:** 1986-12

**Authors:** N. L. Carlsen

## Abstract

During the period 1943-1980 a significant increase in the incidence of neuroblastoma was seen in Denmark. The incidence increased from a level corresponding to that in Finland to a level corresponding to that in the USA, and the increase appears to be continuing. The increase relates to children aged under 5 years, and is most pronounced in infants under 1 year. The incidence in the first year of life has, however, not yet reached the level of the USA. The increase in incidence is most likely a result of improved diagnosis, changes in the social composition of the population, and an increase in environmental carcinogens of importance in the induction of neuroblastomas. The incidence is lower in children of self-employed parents, and higher in infants of mothers aged under 20 or over 34 years. Aside from lower socio-economic circumstances for mothers under 20 years, no specific risk factors were revealed in this study. The observations of a family in which the mother has ganglioneuroma and both daughters have developed neuroblastoma, of a child who suffered from both neuroblastoma and neurofibromatosis von Recklinghausen, and of a significantly higher frequency of infants with signs of multicentric tumours in the offspring of mothers aged under 20 and over 34 years of age, is consistent with the two-hit theory of Knudson et al. (1972).


*Br. J. Cancer* (1986), **54**, 977–988

### Epidemiological investigations on neuroblastomas in Denmark 1943–1980.

N.L.T. Carlsen

*Department of Paediatric Surgery, Rigshospitalet, State University Hospital, DK-2100 Copenhagen, Denmark.*

**Summary** During the period 1943–1980 a significant increase in the incidence of neuroblastoma was seen in Denmark. The incidence increased from a level corresponding to that in Finland to a level corresponding to that in the USA, and the increase appears to be continuing. The increase relates to children aged under 5 years, and is most pronounced in infants under 1 year. The incidence in the first year of life has, however, not yet reached the level of the USA. The increase in incidence is most likely a result of improved diagnosis, changes in the social composition of the population, and an increase in environmental carcinogens of importance in the induction of neuroblastomas.

The incidence is lower in children of self-employed parents, and higher in infants of mothers aged under 20 or over 34 years. Aside from lower socio-economic circumstances for mothers under 20 years, no specific risk factors were revealed in this study.

The observations of a family in which the mother has ganglioneuroma and both daughters have developed neuroblastoma, of a child who suffered from both neuroblastoma and neurofibromatosis von Recklinghausen, and of a significantly higher frequency of infants with signs of multicentric tumours in the offspring of mothers aged under 20 and over 34 years of age, is consistent with the two-hit theory of Knudson *et al.* (1972).

Neuroblastoma, which comprises 7% of all diagnosed cancers in children in the USA, is the most commonly diagnosed neoplasm in newborns, as well as in children between 1 and 12 months (Bader & Miller, 1979; Young & Miller, 1975), and about half of all cases are diagnosed before 2 years of age (Miller *et al.*, 1968; Wilson & Draper, 1974).

The incidence of neuroblastoma displays pronounced geographical differences, and some of this variation can be attributed to factors in the external environment, although ethnic differences in incidence may also point to the importance of genetic factors (Li, 1982; Miller, 1977).

Recent knowledge concerning at least three genetic alterations that are frequently associated with human neuroblastoma suggest that carcinogenesis in neuroblastoma is a multistage process (Schwab *et al.*, 1984), and thus support theories that somatic mutations provoked by external factors lead to the development of malignancy in the cell that is destined to become a cancer cell (Knudson & Strong, 1972; Knudson & Meadows, 1976; Knudson, 1985).

The aim of this study was to investigate whether the epidemiologic rates of neuroblastoma varied in population groups subjected to different environmental pressures – in the broadest sense of the term – and whether the great changes in living conditions of the population as a whole that have taken place in the period 1943–1980, in the form of increasing industrialization and urbanization, have resulted in changes in these rates for neuroblastoma correlated with the calendar year.

Received 10 February 1986; and in revised form 10 July 1986.

### Material and methods

The patient material was collected from two different sources (A & B), as neuroblastomas have not been registered as an entirety in any registry in Denmark during the period of 1943–1977, not even in the Danish Cancer Registry (O.M. Jensen, personal communication).

*A*: All death certificates were examined for children aged less than 15 years, who had died in Denmark and were registered in the Danish National Death Registry at the Danish Institute for Clinical Epidemiology by the underlying cause of death:

*1943–50*: Classified by the Danish code numbers for cancers outside the gastrointestinal tract, the respiratory system, the genital system, the skin, the urinary tract, and osteosarcomas, i.e. 740. + 770.

*1951–68*: Classified by the following code numbers from ICD: 158, 164, 193, 195, 198, 199.

*1969–80*: Classified by the following code numbers from ICD: 158, 163, 192, 195, 199.

A total of 760 children died classified by the

© The Macmillan Press Ltd., 1986

above code numbers in the period of 1943–80, and the hospital records of 316 children, on whom the information in the death certificates did not rule out a possible diagnosis of neuroblastoma, were studied. From this material a total of 186 neuroblastomas were found, of which 173 were histologically verified (group I), while in 13 cases (group II) the case history pointed clearly to neuroblastomas but the histological diagnosis was inconclusive, i.e. suggestive of neuroblastoma, and could not be re-examined (10 cases), or no histological diagnosis had been made and the diagnosis was based on a raised level of urinary VMA excretion (one case) or orbital metastases and tumour abdominis (two cases). One hospital record of a child with the histological diagnosis suggestive of neuroblastoma, had disappeared. One other child who was classified by the above code numbers died at home without having undergone hospitalization; the autopsy revealed neuroblastoma. Finally, in another 15 cases classified by the above code numbers, the nature of the tumour could not be stated, and these cases may also represent neuroblastomas (group III).

*B*: The medical charts of patients below 15 years of age admitted to 9 hospitals in Denmark, which comprise almost all departments of radiation therapy, medical oncology, paediatric surgery, neurosurgery, thoracic surgery, as well as the University Departments of Paediatrics in Denmark during the period of January 1, 1943 to December 31, 1980. The medical records were reviewed for all children that had diagnoses of intra-abdominal or intrathoracic tumour, spinal tumour, CNS tumour, tumour of the peripheral nerves, adrenal tumour, ganglioneuroblastoma, neuroblastoma, sympaticoblastoma, or tumour of unknown origin.

From this source an additional 62 cases were found (excluding 5 patients who were resident outside Denmark when diagnosed), of which 58 were histological verified (group I), whereas in 4 cases (group II) the histological diagnosis was inconclusive and could not be re-examined (two cases) or no histological diagnosis had been made and the diagnosis was based on a raised level of urinary VMA excretion (two cases). All these 62 patients were alive January 1, 1981.

As all the definite or probable neuroblastoma cases who died in the period 1943–80, were also diagnosed in the same period the patient material comprises 232 histologically verified or definite cases (group I), 18 probable but not histologically verified cases (group II), and 15 possible cases (group III) diagnosed during the period 1943–80, and most likely this patient material comprises all the cases of neuroblastoma in childhood in Denmark during that period.

In the 1943–80 birth cohorts a total of 225 definite (group I), 242 definite or probable (group I+II), and 251 definite, probable, or possible (group I+II+III) neuroblastoma cases were diagnosed before the age of 15 years. Sixty-four of the children with definite or probable neuroblastoma were diagnosed in the first year of life and another 11 had undeniable signs of the disease during the first year of life. Thirty of these children died during that first year, whereas another 8 died at older ages.

Neuroblastoma in situ, primary intra-cerebral neuroblastomas, and neuroepitheliomas of the peripheral nerves were not included in this material.

From death certificates and hospital records the place of residence and socio-economic status of the head of family at the time of diagnosis as well as the time of the death of the child were obtained, and this information was classified according to the system used by Danmarks Statistik (Vital statistics). Information concerning the age, place of residence, and socio-economic status of the parents at the time of the child's birth were obtained for all patients with definite or probable neuroblastomas from the birth certificates, and this information was also classified according to the system.

Details were abstracted from the hospital records concerning the time of the child's first symptoms, the time at which diagnosis was made, other diseases suffered by the child or by the mother, particularly during pregnancy, hereditary diseases in the family, and signs of alcohol- or drug-abuse during pregnancy. The data obtained represent the minimum number of these variables.

From the death certificates and birth certificates the father as well as the mother of every child with neuroblastoma in the period of 1943–80 has been identified by name and birthdate, and the proband study thus included all affected near relatives (mother, father, sisters and brothers) in the years 1943–80, but not cousins or more distant family members.

### *The study population*

The mean population of children 0–14 years of age by sex and age, annually 1943–80 was obtained from Danmarks Statistik (Vital statistics), and for the period 1943–70 this mean population by sex and age for capital, provincial towns, and rural areas (from 1970 onward the Reform of Municipalities in 1970 completely altered the definitions of degree of urbanization).

From the information in the National Censuses of 1940, 1950, 1960, 1965, 1970 and 1981, the population of children 0–14 years of age of the 6 main socio-economic groups of the head of family used by Danmarks Statistik could be estimated annually 1943–80. The socio-economic groups, which are used also by the National Board of Health in vital statistics, are as follows: (1) self-employed in agriculture, fishing, etc., (2) other self-employed, (3) salaried employees, (4) manual workers (excluding (5)), (5) manual workers in agriculture, fishing, etc., (6) other, i.e. economically inactive people or industry not stated (including students).

From Danmarks Statistik the annual number of live births, distributed by sex, age and marital stage of mother, and by months of birth was obtained for the period 1943–80. For the period 1943–72 the annual number of live births distributed by the 6 above mentioned socio-economic groups of the head of family was also obtained, and for the period 1943–69 the annual number of live births by domicile in the capital, capital suburbs, provincial towns, the suburbs of provincial towns, other urban settlements (only for the period 1951–69), and rural areas. The age of father has been recorded only for children born in wedlock in Danmarks Statistik 1957–80.

On the basis of these study populations it was possible to calculate:

(i) Rates of incidence and mortality, including age-standardized rates (European standard (Waterhouse *et al.*, 1976)).

(ii) Birth cohort incidence and mortality rates.

These rates were calculated for the definite (group I), the definite or probable (group I+II), and the definite, probable or possible (group I+II+III) neuroblastoma cases individually, and trends in these rates were analysed for significance using logistic regression analysis. Logistic regression analysis was also used to detect significant differences between the rates, whereas the chi-square test was used for testing seasonal variations and genetic factors.

### Results

### *Part I. Rates of mortality and incidence*

During the period 1943–80 there is a significant increase in the annual incidence of neuroblastoma in Denmark for the definite (group I), the definite or probable (group I+II) and even when the possible cases are included (group I+II+III), as well as when these rates are age-standardized ($P<0.001$ in all cases). In contrast, there are no statistically significant variations in mortality rate with calendar year during the period. The number of incident cases and incidence rates are given in Table I.

The trends in the annual age- and sex-specific incidence rates are shown in Figure 1. The

**Figure 1** Average annual sex- and age-specific incidence rates per million in 5-year groups, 1946–80. In each age-group there are 3 curves: The upper comprises the definite, probable and possible cases (I+II+III); the bold curve the definite and probable cases (I+II); and the lower curve, the definite or histologically-verified cases (I). When there are no possible cases the upper and bold curves are superimposable; when there are no probable cases the heavy and lower curves are superimposable. When there are only definite cases all are superimposable giving one bold curve only. The figure shows that the possible (III) and most of the probable (II) cases relate to the younger children, whereas about all cases >4 years are definite (I) cases.

**Table I** Neuroblastoma incidence and mortality trends in Denmark 1943–80. Number of incident cases and average annual incidence and mortality rates per million children in Denmark 1946–80 (definite or probable cases (group I+II).

| | *1946–50* | | | | | *1951–55* | | | | | *1956–60* | | | | | *1961–65* | | | | |
|---|---|---|---|---|---|---|---|---|---|---|---|---|---|---|---|---|---|---|---|---|
| | *Incidence* | | *Mortality* | | | *Incidence* | | *Mortality* | | | *Incidence* | | *Mortality* | | | *Incidence* | | *Mortality* | | |
| *Age* | *No.* | *Rate* | *No.* | *Rate* | *Popul.* | *No.* | *Rate* | *No.* | *Rate* | *Popul.* | *No.* | *Rate* | *No.* | *Rate* | *Popul.* | *No.* | *Rate* | *No.* | *Rate* | *Popul.* |
| 0–1 year | | | | | | | | | | | | | | | | | | | | |
| males | 0 | 0.0 | 0 | 0.0 | 219.6 | 5 | 25.7 | 5 | 25.7 | 194.4 | 3 | 15.5 | 2 | 10.3 | 192.7 | 4 | 19.6 | 3 | 14.7 | 203.4 |
| females | 3 | 14.4 | 2 | 9.6 | 207.9 | 0 | 0.0 | 0 | 0.0 | 183.9 | 2 | 10.9 | 1 | 5.4 | 182.2 | 5 | 25.7 | 2 | 10.3 | 194.1 |
| 1–4 years | | | | | | | | | | | | | | | | | | | | |
| males | 10 | 11.3 | 10 | 11.3 | 878.6 | 7 | 8.7 | 4 | 5.0 | 795.8 | 6 | 7.8 | 7 | 9.2 | 760.5 | 9 | 11.7 | 7 | 9.1 | 763.5 |
| females | 6 | 7.1 | 7 | 8.3 | 836.9 | 5 | 6.5 | 4 | 5.2 | 757.6 | 2 | 2.7 | 2 | 2.7 | 720.0 | 8 | 10.9 | 5 | 6.8 | 730.1 |
| 5–9 years | | | | | | | | | | | | | | | | | | | | |
| males | 4 | 4.5 | 3 | 3.3 | 887.3 | 1 | 0.9 | 1 | 0.9 | 1,085.9 | 3 | 3.0 | 4 | 4.0 | 978.5 | 7 | 7.3 | 6 | 6.3 | 946.7 |
| females | 2 | 2.3 | 1 | 1.1 | 857.0 | 0 | 0.0 | 1 | 0.9 | 1,036.3 | 4 | 4.2 | 5 | 5.3 | 933.5 | 2 | 2.2 | 1 | 1.1 | 899.8 |
| 10–14 years | | | | | | | | | | | | | | | | | | | | |
| males | 2 | 2.5 | 3 | 3.8 | 773.2 | 3 | 3.3 | 2 | 2.2 | 883.4 | 1 | 0.9 | 2 | 1.8 | 1,079.8 | 2 | 2.0 | 2 | 2.0 | 977.3 |
| females | 0 | 0.0 | 0 | 0.0 | 747.7 | 0 | 0.0 | 0 | 0.0 | 852.1 | 2 | 1.9 | 1 | 0.9 | 1,030.8 | 0 | 0.0 | 1 | 1.0 | 933.4 |
| Age-standardized | | | | | | | | | | | | | | | | | | | | |
| males | | 5.5 | | 5.5 | | | 5.7 | | 4.2 | | | 4.6 | | 5.2 | | | 7.7 | | 6.3 | |
| females | | 3.8 | | 3.4 | | | 1.9 | | 1.9 | | | 3.4 | | 3.1 | | | 5.7 | | 3.4 | |
| Total | | 4.7 | | 4.4 | | | 3.8 | | 3.0 | | | 4.1 | | 4.2 | | | 6.7 | | 4.9 | |
| By socio-economic group of head of family: | | | | | | | | | | | | | | | | | | | | |
| Self-employed (group 1+2) | 6 | 2.9 | 6 | 2.9 | 2,028.4 | 7 | 3.5 | 4 | 2.0 | 1,962.8 | 6 | 3.4 | 8 | 4.5 | 1,750.4 | 8 | 5.1 | 7 | 4.4 | 1,563.6 |
| Salaried employees (group 3) | 7 | 8.7 | 6 | 7.5 | 797.4 | 5 | 4.9 | 5 | 4.9 | 1,007.5 | 3 | 2.6 | 3 | 2.6 | 1,117.8 | 11 | 8.4 | 8 | 6.1 | 1,301.5 |
| Manual workers (group 4+5) | 13 | 6.1 | 13 | 6.1 | 2,116.2 | 9 | 3.8 | 8 | 3.4 | 2,335.8 | 14 | 6.0 | 13 | 5.6 | 2,319.4 | 18 | 7.9 | 12 | 5.2 | 2,275.4 |
| Others (group 6) | 1 | 6.5 | 1 | 6.5 | 153.5 | 0 | 0.0 | 0 | 0.0 | 182.8 | 0 | 0.0 | 0 | 0.0 | 165.5 | 0 | 0.0 | 0 | 0.0 | 157.7 |
| Total | 27 | 5.2 | 26 | 5.1 | 5,095.5 | 21 | 3.8 | 17 | 3.0 | 5,488.9 | 23 | 4.2 | 24 | 4.4 | 5,353.1 | 37 | 6.9 | 27 | 5.0 | 5,298.2 |

**Table I** (continued)

| Age | 1966–70 | | | | | 1971–75 | | | | | 1976–80 | | | | | 1946–80 | | | | |
|---|---|---|---|---|---|---|---|---|---|---|---|---|---|---|---|---|---|---|---|---|
| | *Incidence No.* | *Incidence Rate* | *Mortality No.* | *Mortality Rate* | *Popul.* | *Incidence No.* | *Incidence Rate* | *Mortality No.* | *Mortality Rate* | *Popul.* | *Incidence No.* | *Incidence Rate* | *Mortality No.* | *Mortality Rate* | *Popul.* | *Incidence No.* | *Incidence Rate* | *Mortality No.* | *Mortality Rate* | *Popul.* |
| 0–1 year | | | | | | | | | | | | | | | | | | | | |
| males | 6 | 29.6 | 2 | 9.8 | 202.1 | 4 | 21.6 | 1 | 5.4 | 184.5 | 11 | 68.7 | 4 | 25.0 | 160.0 | 33 | 24.3 | 17 | 12.5 | 1,356.7 |
| females | 7 | 36.5 | 3 | 15.6 | 191.5 | 8 | 45.5 | 3 | 17.0 | 175.8 | 5 | 32.8 | 1 | 6.5 | 152.1 | 30 | 23.3 | 12 | 9.3 | 1,287.5 |
| 1–4 years | | | | | | | | | | | | | | | | | | | | |
| males | 12 | 14.4 | 11 | 13.2 | 833.1 | 10 | 13.2 | 7 | 9.3 | 752.6 | 12 | 17.1 | 8 | 11.4 | 700.1 | 66 | 12.0 | 54 | 9.8 | 5,484.2 |
| females | 7 | 8.8 | 7 | 8.8 | 791.8 | 13 | 18.1 | 8 | 11.1 | 714.9 | 16 | 23.8 | 10 | 14.9 | 670.5 | 57 | 10.9 | 43 | 8.2 | 5,221.8 |
| 5–9 years | | | | | | | | | | | | | | | | | | | | |
| males | 6 | 6.2 | 5 | 5.1 | 962.7 | 2 | 1.9 | 3 | 2.9 | 1,032.7 | 3 | 3.1 | 3 | 3.1 | 938.7 | 26 | 3.8 | 25 | 3.6 | 6,832.5 |
| females | 4 | 4.3 | 4 | 4.3 | 921.7 | 3 | 3.0 | 1 | 1.0 | 982.6 | 4 | 4.4 | 2 | 2.2 | 894.4 | 19 | 2.9 | 15 | 2.2 | 6,525.3 |
| 10–14 years | | | | | | | | | | | | | | | | | | | | |
| males | 0 | 0.0 | 0 | 0.0 | 946.8 | 0 | 0.0 | 1 | 1.0 | 967.3 | 1 | 0.9 | 1 | 0.9 | 1,034.0 | 9 | 1.3 | 11 | 1.6 | 6,661.8 |
| females | 0 | 0.0 | 0 | 0.0 | 901.0 | 3 | 3.2 | 3 | 3.2 | 924.8 | 1 | 1.0 | 2 | 2.0 | 984.5 | 6 | 0.9 | 7 | 1.0 | 6,374.3 |
| Age-standardized | | | | | | | | | | | | | | | | | | | | |
| males | | 8.2 | | 6.1 | | | 6.0 | | 4.3 | | | 11.2 | | 6.4 | | | 6.9 | | 5.4 | |
| females | | 6.5 | | 5.0 | | | 10.5 | | 5.7 | | | 11.0 | | 6.0 | | | 6.0 | | 4.0 | |
| Total | | 7.4 | | 5.5 | | | 8.2 | | 5.0 | | | 11.1 | | 6.3 | | | 6.4 | | 4.7 | |
| By socio-economic group of head of family: | | | | | | | | | | | | | | | | | | | | |
| Self-employed (group 1+2) | 7 | 5.0 | 5 | 3.5 | 1,391.3 | 5 | 4.0 | 2 | 1.6 | 1,221.5 | 8 | 7.5 | 4 | 3.7 | 1,063.5 | 47 | 4.2 | 36 | 3.2 | 10,981.5 |
| Salaried employees (group 3) | 18 | 11.1 | 13 | 8.0 | 1,618.6 | 18 | 9.4 | 10 | 5.2 | 1,910.0 | 26 | 12.1 | 16 | 7.4 | 2,142.0 | 88 | 8.8 | 61 | 6.1 | 9,894.8 |
| Manual workers (group 4+5) | 15 | 6.5 | 13 | 5.6 | 2,307.0 | 20 | 8.8 | 14 | 6.1 | 2,266.3 | 14 | 6.5 | 10 | 4.6 | 2,135.5 | 103 | 6.5 | 83 | 5.2 | 15,755.6 |
| Others (group 6) | 2 | 9.9 | 1 | 4.9 | 201.4 | 0 | 0.0 | 0 | 0.0 | 257.8 | 5 | 16.7 | 1 | 3.3 | 298.8 | 8 | 5.6 | 4 | 2.8 | 1,417.5 |
| Total | 42 | 7.6 | 32 | 5.7 | 5,518.3 | 43 | 7.6 | 27 | 4.7 | 5,655.6 | 53 | 9.3 | 31 | 5.4 | 5,639.8 | 246 | 6.4 | 184 | 4.8 | 38,049.4 |

No. = number. Popul. = population in thousands. Note that the estimated populations by socio-economic groups differ from the mean population due to rounding. Standard population = European standard of children below 15 years of age in millions.

F

incidence is significantly higher for children aged less than 5 years ($P<0.001$), and the increase in incidence during the study period could be ascribed solely to the increase in incidence in this age group, as there are no significant variations in incidence rate for children 5 years of age or more with respect to calendar year ($P=0.304$). In the age group under 5 years, the incidence is significantly higher for infants aged below 1 year ($P<0.001$). Throughout the entire period the mortality rate is significantly higher in children under 5 years ($P<0.001$), but is not different for children under 1 year and children from 1 to 4 years. The number of incident cases and incidence rates are given in Table I.

There is no significant difference between sexes with respect to calendar year. However, if age is taken into consideration, the difference between the sexes is significant (sex: $P=0.02$, age: $P<0.001$, interaction between sex and age: $P=0.35$). In contrast, the mortality rate is significantly higher for boys than for girls when the probable and possible cases are included ($P=0.02$, respectively $P=0.03$), but not when only the definite cases are considered ($P=0.11$). The number of incident cases and incidence rates are given in Table I.

The incidence rates (not age-standardized) are significantly lower for children of self-employed parents (socio-economic groups 1 and 2) compared with the rest of the children ($P=0.027$), whereas the differences in mortality rates between children of self-employed parents and the others are not significant ($P=0.11$). The number of incident cases and incidence rates are given in Table I.

No significant differences are found in age-standardized incidence or mortality rates with respect to residence at time of first symptom, diagnosis, or death.

No significant differences are found in seasonal variations with respect to month of the first symptom, the diagnosis, or death.

*Part II. Birth cohort mortality and incidence rates.*

During the period 1943–80 there is a significant increase in birth cohort incidence of childhood neuroblastoma for the cohorts born between 1943 and 1972 ($P<0.001$), and the increase seems to be continuing for the cohorts born 1973–77 despite the lack of 15-year observations of the cohorts. In contrast, there are no significant changes in the mortality rates for the birth cohorts 1943–72 when the probable and possible cases are included ($P=0.17$, respectively $P=0.60$), whereas there is a significant increase in birth cohort mortality for the definite cases ($P=0.04$). During the same period the increase in incidence in the first year of life is also significant for the cohorts born between 1943 and 1980 ($P<0.01$), whereas the mortality in the first year of life did not change significantly, The number of incident cases and incidence rates are given in Table II.

The birth cohort incidence rates are significantly higher for children of mothers aged under 20 years or over 34 years, than for children of mothers aged 20–34 years ($P=0.02$) for the cohorts born between 1943 and 1972. Similarly, there are significantly higher birth cohort mortality rates when the maternal age is below 20 or above 34 years at birth, compared with maternal age between 20 and 34 years ($P=0.01$). The number of incident cases and incidence rates are given in Table II.

The birth cohort incidence rates are lower for children born to self-employed parents (socio-economic groups 1 and 2) 1943–72 than for other children, but the differences are not statistically significant ($P=0.07$), contrary to the birth cohort mortality rates which are significantly lower for children of self-employed parents ($P=0.050$). However, when maternal age is taken into consideration, the birth cohort incidence rates are also significantly lower for children born to self-employed parents (socio-economic group: $P=0.022$, maternal age: $P=0.056$). The number of incident cases and incidence rates are given in Table II.

Further analysis of the correlation between maternal age and socio-economic group reveals that the incidence for children diagnosed during the first year of life is significantly higher when the maternal age is below 20 years (maternal age: $P=0.05$, socio-economic group: $P=0.08$), whereas the offspring of mothers aged above 34 years has the lowest incidence of neuroblastoma during the first year of life. In contrast, the incidence for children 1–14 years of age at diagnosis is significantly higher when the maternal age is above 34 years (maternal age: $0.025<P<0.05$, socio-economic group: $P=0.24$), whereas the incidence after the first year of life is not significantly different for children of mothers aged below 20 years and mothers aged 20–34 years ($P>0.10$). However, both maternal age and socio-economic group are independent in this analysis.

There is a very close correlation between paternal and maternal age both in the neuroblastoma material and in the study population, and in fact no independent assessment of age effect could be made for paternal *versus* maternal age. Thus, the possibility exists that the maternal age effect in this study could be due to a paternal age effect.

There are no significant differences in birth cohort incidence or mortality rates for the cohorts born between 1943 and 1969 with respect to domicile at birth. No significant seasonal variations in birth cohort incidence or mortality rates are found with respect to months of birth 1943–72.

*Part III. Prenatal and genetic factors*

*Coincidence with the so-called neurocristopathies* (*Bolande, 1974*): One patient in this material had both neuroblastoma and neurofibromatis von Recklinghausen, which is more than the expected coincidence due to chance of 0.09 children in Denmark during the years 1943–80, or 0.5 at the 95% confidence limits [as the incidence of von Recklinghausen's disease is 1 per 2,5–3,300 live births (Waardenburg *et al.*, 1963)]. No cases with neuroblastoma and any other of the neurocristopathies were found.

*Family prevalence of neuroblastoma:* The mother of one child with neuroblastoma in this material had herself been operated at age 7 and 9 years for ganglioneuroma in the thorax and pelvis respectively, and at age 19 years was re-operated upon for pelvic ganglioneuroma. In 1984 the younger sister to the child in the study material was diagnosed as also having neuroblastoma; thus in this family the mother, who has multiple ganglioneuromas, has given birth to two girls who at age 2 and 8 years respectively were diagnosed as having neuroblastoma (the mother was 25 and 31 years of age at birth, respectively). This is the only case of familial neuroblastoma in Denmark during the study period.

*Sex*: The male/female ratio for the definite or probable cases born 1943–80 is 1.10, which did not differ significantly from the expected ratio of 1.06 in Denmark during this period.

No cases of maternal epilepsy were recorded, and there was no information in the hospital records of other hereditary diseases, or alcohol- or drug-abuse during pregnancy. The number of complicated pregnancies recorded in this study does not seem to differ from the frequency one could expect. However, the data recorded in the hospital records represent the minimum number of these complications. Three of the patients had cerebral palsy; 2 of these were due to neonatal asphyxia and 1 resulted from meningitis.

Eight cases with multicentric primary tumours according to Knudson & Strong (1972) with involvement of both adrenals or the adrenal and the paravertebral sympathetic ganglia, had signs of the disease during the first year of life. This figure represents the minimum number, since bilateral adrenal involvement is diagnosed only at autopsy. It is notable that 4 of these 8 patients (3 of the 5 diagnosed in the first 3 months of life) were born to mother below 20 or above 34 years of age, which represent 4/14 (or 3/14) of the children born to mothers in these age groups compared with only 4/61 (or 2/61) of the children born to mothers between 20 and 34 years of age ($P=0.04$ in both cases) in the patient material of neuroblastoma in the first year of life.

### Discussion

During the period covered by this study, a significant increase in incidence was observed in Denmark, from a level corresponding to that in Finland (Teppo *et al.*, 1975) to a level corresponding to that in the USA (Young & Miller, 1975), and the increase appears to be continuing. This increase results solely from an increase in incidence for children aged under 5 years, and is most pronounced in children under 1 year. A corresponding increase was observed in the birth cohort incidence, an increase which, despite the lack of 15-year observations of all birth cohorts, appears to continue in the period 1973–77. The birth cohort incidence in the first year of life has, however, not yet reached the level of the USA (Bader & Miller, 1979). On the other hand, neither the mortality nor the birth cohort mortality rates have changed significantly during the period in question, though the former has risen somewhat. During the same period the long-term survival has gradually improved in Denmark from 0% during the period 1943–50 to 32% during the period 1971–80. The better survival obtained from decade to decade was due to a combination of a higher frequency of lower stages of the disease, younger ages at diagnosis, and multi-modal treatment including chemotherapy (Carlsen *et al.*, 1986). In Sweden (Erichsson *et al.*, 1978) as well as in Denmark (Olsen & Scheibel, 1984) a significant increase in incidence of tumours of the nervous system (including neuroblastomas) in childhood has been reported, and in Sweden the rise in incidence of neuroblastoma was also significant (Erichsson *et al.*, 1978). In contrast, the incidence of neuroblastoma has remained fairly constant in Manchester, England, throughout the period 1954–77 (Birch *et al.*, 1980).

Despite the fact that all the known cases of deaths from neuroblastoma amongst patients at the 9 hospitals investigated in this study have been found again under the code numbers examined, is the increase in incidence demonstrated in this study genuine? In the period in question the code numbers in the Danish National Death Registry have been changed twice, and the use of autopsy in cases of death in children has also become more frequent. Furthermore, diagnostic techniques have undoubtedly been improved, for example by the introduction of the urinary VMA excretion as a diagnostic tool in the sixties. The frequency of autopsy for death statistics is significant (Juel, 1981); however, it seems difficult to believe that the

**Table II** Neuroblastoma birth cohort incidence and mortality trends 1943–80. Number of incident cases and average annual incidence and mortality rates per 100,000 live births 1943–77 (definite or probable cases (group I + II)).

| | 1943–47 | | | | | 1948–52 | | | | | 1953–57 | | | | | 1958–62 | | | | |
|---|---|---|---|---|---|---|---|---|---|---|---|---|---|---|---|---|---|---|---|---|
| | *Incidence* | | *Mortality* | | | *Incidence* | | *Mortality* | | | *Incidence* | | *Mortality* | | | *Incidence* | | *Mortality* | | |
| *Age* | *No.* | *Rate* | *No.* | *Rate* | *Popul.* | *No.* | *Rate* | *No.* | *Rate* | *Popul.* | *No.* | *Rate* | *No.* | *Rate* | *Popul.* | *No.* | *Rate* | *No.* | *Rate* | *Popul.* |
| Total | 27 | 5.9 | 26 | 5.7 | 457.8 | 21 | 5.3 | 21 | 5.3 | 397.9 | 29 | 7.6 | 25 | 6.5 | 383.5 | 28 | 7.4 | 25 | 6.6 | 378.9 |
| 1 year of life | 4 | 0.9 | 3 | 0.7 | | 1 | 0.3 | 1 | 0.3 | | 8 | 2.1 | 6 | 1.6 | | 6 | 1.6 | 4 | 1.1 | |
| Maternal age | | | | | | | | | | | | | | | | | | | | |
| <20 y | 2 | 7.6 | 2 | 7.6 | 26.4 | 0 | 0.0 | 0 | 0.0 | 28.1 | 4 | 12.5 | 4 | 12.5 | 32.0 | 2 | 5.2 | 1 | 2.6 | 38.8 |
| 20–29 y | 17 | 6.6 | 17 | 6.6 | 256.9 | 14 | 6.0 | 14 | 6.0 | 232.9 | 18 | 7.8 | 14 | 6.0 | 231.9 | 19 | 8.0 | 18 | 7.6 | 237.4 |
| 30–34 y | 4 | 3.9 | 3 | 3.0 | 101.5 | 3 | 3.8 | 3 | 3.8 | 78.6 | 3 | 4.1 | 3 | 4.1 | 73.1 | 2 | 3.2 | 2 | 3.2 | 63.2 |
| >34 y | 4 | 5.3 | 4 | 5.3 | 75.0 | 4 | 6.7 | 4 | 6.7 | 59.9 | 4 | 8.4 | 4 | 8.4 | 47.8 | 5 | 12.7 | 4 | 10.1 | 39.5 |
| Socio-economic group | | | | | | | | | | | | | | | | | | | | |
| Self-employed (group 1 + 2) | 5 | 3.4 | 5 | 3.4 | 147.4 | 8 | 6.3 | 8 | 6.3 | 126.0 | 8 | 7.2 | 6 | 5.4 | 110.5 | 4 | 3.9 | 3 | 2.9 | 102.6 |
| Salaried employees (group 3) | 4 | 5.8 | 4 | 5.8 | 69.2 | 3 | 4.6 | 3 | 4.6 | 65.7 | 6 | 8.1 | 5 | 6.7 | 74.2 | 7 | 8.1 | 6 | 6.9 | 86.4 |
| Manual workers (group 4 + 5) | 18 | 7.7 | 17 | 7.2 | 234.9 | 10 | 5.0 | 10 | 5.0 | 201.6 | 14 | 7.2 | 13 | 6.7 | 194.5 | 17 | 9.4 | 16 | 8.9 | 180.0 |
| Others (group 6) | 0 | 0.0 | 0 | 0.0 | 8.3 | 0 | 0.0 | 0 | 0.0 | 6.3 | 1 | 16.1 | 1 | 16.1 | 6.2 | 0 | 0.0 | 0 | 0.0 | 10.3 |
| Total | 27 | 5.9 | 26 | 5.7 | 459.8 | 21 | 5.3 | 21 | 5.3 | 399.6 | 29 | 7.5 | 25 | 6.5 | 385.4 | 28 | 7.4 | 25 | 6.6 | 379.3 |

**Table II** (continued)

| Age | 1963–67 | | | | | 1968–72* | | | | | 1973–77* | | | | | 1943–73 | | | | |
|---|---|---|---|---|---|---|---|---|---|---|---|---|---|---|---|---|---|---|---|---|
| | Incidence | | Mortality | | | Incidence | | Mortality | | | Incidence | | Mortality | | | Incidence | | Mortality | | |
| | No. | Rate | No. | Rate | Popul. | No. | Rate | No. | Rate | Popul. | No. | Rate | No. | Rate | Popul. | No. | Rate | No. | Rate | Popul. |
| Total | 43 | 10.2 | 31 | 7.4 | 421.3 | 41 | 11.2 | 28 | 7.6 | 367.5 | 41 | 12.0 | 19 | 5.5 | 342.4 | 189 | 7.9 | 156 | 6.5 | 2,406.9 |
| 1 year of life | 13 | 3.1 | 5 | 1.2 | | 11 | 3.0 | 5 | 1.4 | | 12 | 3.5 | 1 | 0.3 | | 43 | 1.8 | 24 | 1.0 | |
| Maternal age | | | | | | | | | | | | | | | | | | | | |
| <20 y | 10 | 20.3 | 8 | 16.3 | 49.2 | 6 | 19.0 | 4 | 12.7 | 31.6 | | | | | | 24 | 11.6 | 19 | 9.2 | 206.1 |
| 20–29 y | 22 | 7.9 | 15 | 5.4 | 278.0 | 28 | 10.6 | 19 | 7.2 | 263.0 | | | | | | 118 | 7.9 | 97 | 6.5 | 1,500.1 |
| 30–34 y | 5 | 8.3 | 2 | 3.3 | 60.1 | 3 | 5.9 | 1 | 2.0 | 51.2 | | | | | | 20 | 4.7 | 14 | 3.3 | 427.7 |
| >34 y | 6 | 17.6 | 6 | 17.6 | 34.0 | 4 | 18.3 | 4 | 18.3 | 21.8 | | | | | | 27 | 9.7 | 26 | 9.4 | 278.0 |
| Socio-economic group | | | | | | | | | | | | | | | | | | | | |
| Self-employed (group 1+2) | 5 | 5.9 | 4 | 4.8 | 84.1 | 3 | 5.9 | 2 | 3.9 | 51.1 | | | | | | 33 | 5.3 | 28 | 4.5 | 621.7 |
| Salaried employees (group 3) | 15 | 12.1 | 10 | 8.1 | 124.0 | 18 | 14.3 | 12 | 9.5 | 126.3 | | | | | | 53 | 9.7 | 40 | 7.3 | 545.8 |
| Manual workers (group 4+5) | 21 | 10.6 | 16 | 8.1 | 197.8 | 17 | 10.1 | 11 | 6.6 | 167.7 | | | | | | 97 | 8.2 | 83 | 7.1 | 1,176.5 |
| Others (group 6) | 2 | 13.3 | 1 | 6.7 | 15.0 | 3 | 13.3 | 3 | 13.3 | 22.5 | | | | | | 6 | 8.7 | 5 | 7.3 | 68.6 |
| Total | 43 | 10.2 | 31 | 7.4 | 420.9 | 41 | 11.2 | 28 | 7.6 | 367.6 | | | | | | 189 | 7.8 | 156 | 6.5 | 2,412.6 |

No. = number. Popul. = population in thousands. Note that the populations by maternal age and socio-economic group differ from the birth cohort populations due to rounding. *Indicates lack of 15-year observation of the cohorts.

ominous symptoms of cancer could have been overlooked during the first part of the study period, so that children were incorrectly registered as dying of non-neoplastic diseases, even though the possibility cannot be dismissed (Schottenfeld, 1981; Young & Miller, 1975). The present study demonstrates that neuroblastoma cases which might have been wrongly attributed to death by other cancers, should be sought amongst children who died before their 5th year. However, only the incidence of tumours of the nervous system in childhood has risen during the period 1943–80 in Denmark (Olsen & Scheibel, 1984). Neuroblastoma cases with spontaneous cure or cure obtained in other hospitals than those investigated might have escaped ascertainment in this study; however, the rate of spontaneous regression or maturation of clinically overt disease has been estimated to be 8 per cent of cases (Evans *et al.*, 1976), and this frequency obviously cannot influence the present estimates of the true trends in incidence significantly. The observation of a continuing rise in incidence in recent years despite the lack of noteworthy improvement in diagnosis, and despite no further centralization of treatment (Carlsen *et al.*, 1985), support the conjecture that the rise in incidence is real. It is therefore believed that the present estimates reflect the true trends in incidence of childhood neuroblastoma in Denmark during the period 1943–80. Some of this rise in incidence would be consistent with the suggestion that environmental carcinogens of importance in the induction of neuroblastoma may have increased (Schottenfeld, 1981).

The investigation revealed that the incidence as well as the mortality is significantly lower for the children of self-employed parents than for children in other socio-economic groups. In Denmark the living conditions for children born to self-employed parents are generally the best and the percentage of extra-marital births is very low in the group of self-employed. Social class factors have also been revealed for other types of childhood neoplasms (Gutensohn *et al.*, 1982; Ramot & Magrath, 1982; Schottenfeld, 1981). During the study period the percentage of children of self-employed parents fell from 41% to 19%. However, changes in the composition of the population cannot account for the observed increase on their own.

The study revealed that the birth cohort incidence as well as the birth cohort mortality are significantly higher for children of mothers aged under 20 or over 34 years. Further analysis suggests that the significantly higher birth cohort incidence when maternal age is under 20 years is due to an over-representation of neuroblastomas in the first year of life. In Denmark the social stresses in this maternal age group are higher than in other maternal age groups (high frequency of extra-marital births, lower socio-economic class). One may speculate that some of the children born to mothers under 20 years have been highly exposed to carcinogens in early gestational life, since the incidence in the first year of life is higher (Miller, 1977; Rice, 1973; Schottenfeld, 1981). However, aside from lower socio-economic circumstances, this study revealed no specific risk factors [as for example a higher alcohol consumption during pregnancy (Seeler *et al.*, 1979)].

The significantly higher birth cohort incidence for children of mothers over 34 years of age is due to an over-representation of neuroblastomas diagnosed after the first year of life. In this age group the percentage of illegitimate births and the percentage of children born to self-employed parents did not differ from the maternal age group of 20–34 years. According to the theory of Knudson & Strong (1972), one might have expected that the offspring of mothers over 34 years of age would have the highest incidence of neuroblastoma in the first year of life, since women older than 34 have a greater risk of developing gonadal chromosomal alterations. Several other studies have demonstrated an increased risk of childhood cancer with advanced maternal age, whereas others found no such effect (Daling *et al.*, 1984). This study revealed a significantly higher frequency of children with signs of multicentric primary tumours as defined by Knudson and Strong (1972) in infants born to mothers under 20 and over 34 years of age, but the figures are small. In relation to these findings, Evans' (1965) report of 5 congenital cases is interesting. Three of the tumours had bilateral adrenal involvement, and the mother of one of these three infants was 40 years of age. In other reports of congenital cases in the literature the age of the mother is not stated.

Only one case of familial neuroblastoma was observed in this study, and only one child with both neuroblastoma and neurofibromatosis von Recklinghausen, whereas no cases with neuroblastoma and any other of the so-called neurocristopathies were found (Bolande, 1974).

To explain the large geographical differences in neuroblastoma incidence, Knudson and Meadows (1976) suggested that children destined to develop neuroblastoma might also suffer higher perinatal mortality (this hypothesis is discussed by Miller, 1977). An exponential fall has in fact occurred in mortality during the first year of life in Denmark, from $4{,}900 \times 10^{-5}$ live births in 1941–45 to $900 \times 10^{-5}$ live births in 1976–80 and the increase in neuroblastoma incidence might thus solely be explained as the result of a decrease in neonatal mortality during the period of study, assuming this theory.

### Conclusion

Only a few reliable population-based studies on trends in incidence of childhood neuroblastoma have been carried out (Birch *et al.*, 1980, Erichsson *et al.*, 1978; Teppo *et al.*, 1975; Young & Miller, 1975), since most international data are presented using classifications based on site (Clemmesen, 1964–5; Waterhouse *et al.*, 1976), and neuroblastomas might be represented at more than 10 sites. This study which most likely comprises all the cases of neuroblastoma in Denmark 1943–80, revealed a significant increase in incidence during the study period. This increase in incidence is most likely a result of better diagnosis, changes in the social composition of the population, and possibly unidentified environmental agents. The incidence is lower in children of self-employed parents, and higher in children of mothers aged under 20 or over 34 years. Infants of mothers younger than 20 have a higher risk of developing neuroblastomas in the first year of life, and these mothers have lower socio-economic circumstances in Denmark. Thus, the higher incidence could be a result of greater environmental stress during early gestational life. However, no specific risk factors were revealed. Children born to mothers over 34 years have the lowest incidence during the first year of life, suggesting that a possible hereditary mutation may play its role later in life, but these considerations are speculative, since most neuroblastomas are most likely congenital (Birch *et al.*, 1980; Carlsen *et al.*, 1985; Wilson & Draper, 1974).

A significantly higher frequency of infants with signs of multicentric primary tumours was found in the offspring of mothers under 20 and over 34 years of age, but the figures are small. This finding, the observation of a family in which the mother has ganglioneuroma and both daughters have developed neuroblastoma, and the observation of a child with both neuroblastoma and neurofibromatosis von Recklinghausen, is consistent with the two-hit theory of Knudson *et al.* (Knudson & Strong, 1972; Knudson & Meadows, 1976; Knudson, 1985) that some neuroblastomas might have a hereditary component in the form of an inherited first mutation.

I thank Mrs. V. Rosenkrantz, the National Board of Health, for assistance in obtaining death certificates, and Mrs K. Frandsen, Danmarks Statistik, for assistance in obtaining the study population. I thank the Danish Ministry for Church Affairs and all its registrars for assistance in obtaining copies of birth certificates. Senior physicians at almost all Danish hospitals are thanked for placing hospital records at my disposal, with special thanks to the many medical secretaries who located them in hospital archives. Drs K. Hou-Jensen, (Finsen Institute), I. Tygstrup, and K. Ørnvold (Rigshospitalet) are thanked for assistance in re-examining the histological preparations. Dr. H. Bay-Nielsen (Glostrup Hospital) is thanked for permission to make comparison with his material of childhood neoplasms, and the Danish Cancer Registry for permission to make comparison with the Registry's neuroblastoma material for the period of 1978 to 1980, in which period neuroblastoma cases have been registered there as an entirety. I thank Prof. M. Hauge (University of Odense) for advice on problems of heredity. In particular I thank Mette Madsen, and Ib J. Christensen (Finsen Laboratory), who gave invaluable assistance with the statistical calculations. This investigation was in part supported by a grant from the Danish Medical Research Council (grant no. 5.52.12.77).

### References

BADER, J.L. & MILLER, R.W. (1979). US cancer incidence and mortality in the first year of life. *Am. J. Dis. Child* **133,** 157.

BIRCH, J.M., MARSDEN, H.B. & SWINDELL, R. (1980). Incidence of malignant disease in childhood: A 24-year review of the Manchester Children's Tumour Registry data. *Br. J. Cancer* **42,** 215.

BOLANDE, R.P. (1974). The neurocristopathies – a unifying concept of disease arising in neural crest maldevelopment. *Hum. Pathol.* **5,** 409.

CARLSEN, N.L.T., SCHROEDER, H., BRO, P.V. & 4 others (1985). Neuroblastomas treated at the four major child oncologic clinics in Denmark 1943–1980: An evaluation of 180 cases. *Med. Pediatr. Oncol.* **13,** 180.

CARLSEN, N.L.T., CHRISTENSEN, I.J., SCHROEDER, H. & 5 others (1986). Prognostic factors in neuroblastomas treated in Denmark during the period of 1943–1980. *Cancer* (in press).

CLEMMESEN, J. (1964–65). Statistical studies in the aetiology of malignant neoplasms. *Acta. Pathol. Microbiol. Scand.*, suppl. **174,** I–II.

DALING, J.R., STARZYK, P., OLSHAN, A.F. & WEISS, N.S. (1984). Birth weight and the incidence of childhood cancer. *J. Natl Cancer Inst.* **72,** 1039.

ERICHSSON, J.L.-E., KARNSTRÖM, L. & MATTSSON, B. (1978). Childhood cancer in Sweden, 1958–1974. I. Incidence and mortality. *Acta Paediatr. Scand.* **67,** 425.

EVANS, A.E., GERSON, J. & SCHNAUFER, L. (1976). Spontaneous regression of neuroblastoma. *Natl Cancer Inst. Monogr.* **44,** 49.

EVANS, A.R. (1965). Congenital neuroblastoma. *J. Clin. Pathol.* **18,** 54.

GUTENSOHN, N.M. & SHAPIRO, D.S. (1982). Social class risk factors among children with Hodgkin's disease. *Int. J. Cancer* **30,** 433.

JUEL, K. (1981). The significance of the frequency of autopsy for statistics of the causes of death. *Ugeskr. Læg.* **143,** 2669.

KNUDSON, A.G. & STRONG, L.C. (1972). Mutation and cancer. Neuroblastoma and pheochromocytoma. *Amer. J. Hum. Genet.* **24,** 514.

KNUDSON, A.G. & MEADOWS, A.T. (1976). Developmental genetics of neuroblastoma. *J. Natl Cancer Inst.* **57,** 675.

KNUDSON, A.G. (1985). Hereditary cancer, oncogenes, and antioncogenes. *Cancer Res.* **45,** 1437.

LI. F.P. (1982). Cancer in children. In *Cancer Epidemiology and Prevention,* Scottenfeld, D. & Fraumeni J.F. (eds), p. 1012. W.B. Saunders: Philadelphia.

MILLER, R.W., FRAUMENI, J.F. & HILL, J.A. (1968). Neuroblastoma: Epidemiologic approach to its origin. *Am. J. Dis. Child* **115,** 253.

MILLER, R.W. (1977). Ethnic differences in cancer occurrence: Genetical and environmental influences with particular reference to neuroblastoma. In *Genetics of Human Cancer,* Mulvihill, J.J., *et al.* (eds), p. 1. Raven Press: New York.

OLSEN, J.H. & SCHEIBEL, E. (1984). Cancer i barnealderen i Danmark 1943–1980 (Incidence of childhood cancer in Denmark 1943–1980). *Ugeskr. Læg.* **146,** 3228.

RAMOT, B. & MAGRATH, I. (1982). Annotation. *Br. J. Haematol.* **52,** 183.

RICE, J.M. (1973). An overview of transplacental chemical carcinogenesis. *Teratology* **8,** 113.

SCHOTTENFELD, D (1981). The epidemiology of cancer: An overview. *Cancer* **47,** 1095.

SCHWAB, M., VARMUS, H.E., BISHOP, J.M. & 5 others (1984). Chromosome localization in normal human cells and neuroblastomas of a gene related to c-*myc*. *Nature* **308,** 288.

SEELER, R.A., ISRAEL, J.N., ROYAL, J.E., KAYE, C.I., RAO, S. & ABULABAN, M. (1979). Ganglioneuroblastoma and fetal hydantoin-alcohol syndromes. *Pediatrics* **63,** 524.

TEPPO, L., SALONEN, T. & HAKULINEN, T. (1975). Incidence of childhood cancer in Finland. *J. Natl Cancer Inst.* **55,** 1065.

WAARDENBURG, P.J., FRANCESCHETTI, A. & KLEIN, D. (1963). *Genetics and Opthalmology,* p. 1355. Blackwell Scientific Publications: Oxford.

WATERHOUSE, J., MUIR, C., CORREA, P. & POWEL, J. (eds) (1976). *Cancer Incidence in Five Continents,* Vol. III, IARC Scientific Publications No. 15, Lyon.

WILSON, L.M.K. & DRAPER, G.J. (1974). Neuroblastoma, its natural history and prognosis: A study of 487 cases. *Br. Med. J.* **2,** 301.

YOUNG, J.L. & MILLER, R.W. (1975). Incidence of malignant tumours in US children. *J. Pediatr.* **86,** 254.